# Dung beetles of Azorean cattle-grazed pasturelands - data of the DUNGPOOL project

**DOI:** 10.3897/BDJ.13.e163289

**Published:** 2025-10-01

**Authors:** Almudena Duenas-Rojas, Eva Cuesta, Laurine M. Parmentier, Abrão Leite, Paulo A. V. Borges, Ana M. C. Santos

**Affiliations:** 1 Centro de Investigación en Biodiversidad y Cambio Global (CIBC-UAM), Universidad Autónoma de Madrid, Calle Darwin 2, 28049, Madrid, Spain Centro de Investigación en Biodiversidad y Cambio Global (CIBC-UAM), Universidad Autónoma de Madrid, Calle Darwin 2, 28049 Madrid Spain; 2 Terrestrial Ecology Group (TEG-UAM), Departamento de Ecología, Universidad Autónoma de Madrid, Calle Darwin 2, Edificio de Biología, 28049, Madrid, Spain Terrestrial Ecology Group (TEG-UAM), Departamento de Ecología, Universidad Autónoma de Madrid, Calle Darwin 2, Edificio de Biología, 28049 Madrid Spain; 3 University of the Azores, cE3c- Centre for Ecology, Evolution and Environmental Changes/Azorean Biodiversity Group, CHANGE – Global Change and Sustainability Institute, School of Agricultural and Environmental Sciences, Rua Capitão João d´Ávila, Pico da Urze, 9700-042, Angra do Heroísmo, Azores, Portugal University of the Azores, cE3c- Centre for Ecology, Evolution and Environmental Changes/Azorean Biodiversity Group, CHANGE – Global Change and Sustainability Institute, School of Agricultural and Environmental Sciences, Rua Capitão João d´Ávila, Pico da Urze, 9700-042 Angra do Heroísmo, Azores Portugal; 4 Rua Fernando Pessoa, n. 99 2765-483, Estoril, Portugal Rua Fernando Pessoa, n. 99 2765-483 Estoril Portugal; 5 IUCN SSC Atlantic Islands Invertebrate Specialist Group, 9700-042, Angra do Heroísmo, Azores, Portugal IUCN SSC Atlantic Islands Invertebrate Specialist Group, 9700-042 Angra do Heroísmo, Azores Portugal; 6 IUCN SSC Species Monitoring Specialist Group, 9700-042, Angra do Heroísmo, Azores, Portugal IUCN SSC Species Monitoring Specialist Group, 9700-042 Angra do Heroísmo, Azores Portugal

**Keywords:** Azores Islands, Faial Island, Pico Island, Terceira Island, Coleoptera, Scarabaeidae, dung beetles, occurrence event, pastures, pitfall traps, sampling event

## Abstract

**Background:**

The data we present are part of the project DUNGPOOL, which aims to evaluate the effects of the species pool, community assembly processes and increasing temperatures on the local diversity and ecosystem functions performed by dung beetles in island and mainland cattle-grazed pasturelands. By combining replicated field experiments on the Iberian Peninsula with parallel work on three mid-Atlantic islands (Pico, Faial and Terceira, Azores), the project exploits the natural contrast between a species-rich mainland pool and the species-poor, largely exotic island pools, to test explicit biodiversity–ecosystem-function (BEF) hypotheses across spatial scales.

From June to July 2024, dung beetles were sampled in 84 locations of the three islands of the Azores Archipelago (Portugal), using 306 pitfall traps baited with fresh cow dung.

**New information:**

A total of 1,141 dung beetle individuals were recorded across these islands: 18 individuals in Pico Island, 480 individuals in Faial Island and 643 individuals in Terceira Island. These individuals were distributed amongst four species from the family Scarabaeidae (Insecta, Coleoptera): *Calamosternus granarius* (Linnaeus, 1767), *Labarrus lividus* (Olivier, 1789), *Onthophagus taurus* (Schreber, 1759) and *Onthophagus medius* (Kugelann, 1792). The species *O. medius* is a new record for this Archipelago and we propose that previous historical records of *Onthophagus vacca* (Linnaeus, 1767) should now be considered a regional synonym of *Onthophagus medius*.

## Introduction

Dung beetles (Coleoptera, Scarabaeoidea) are known for their coprophagous habits. They are classified into three functional groups based on their behavioural strategies: telecoprids, species that roll dung balls and bury them at a distance from the main dung pile; paracoprids, those that make galleries in the soil and bury the dung underneath the main dung pile, in which they bury small pieces of dung; and endocoprids, those that feed in the dung. As a result of these habits, dung beetles perform different ecological functions that provide important ecosystem services (Nichols et al. 2008), like the decomposition and burial of dung and secondary dispersal of seeds (deCastro‐Arrazola et al. 2020, Kadiri et al. 2022, deCastro‐Arrazola et al. 2023), amongst others. Human activities associated with landscape transformation and habitat fragmentation are a serious threat to dung beetles (Noriega et al. 2021, Lumaret et al. 2022). The intensification of livestock grazing alters the natural conditions of the soil and, therefore, their habitat and diversity (Noriega et al. 2023). On the other hand, the use of antiparasitics in livestock, such as ivermectins, also affects the diversity and ecosystem functions of dung beetles (Verdú et al. 2018, Ambrožová et al. 2021).

Many dung beetles are associated with cattle-grazed pasturelands, a land use of high cultural and economic importance in the Azorean Islands, Portugal (Borges et al. 2020), an isolated archipelago of volcanic origin that is located in the North Atlantic and is part of the Macaronesia Region. The Azores were discovered in the 15^th^ century by the Portuguese and, since human colonisation, native habitats have been widely modified (Borges et al. 2020).

Humans introduced livestock in these islands and because of its expansion, many native habitats were replaced by pastures for cattle grazing. The dung beetles present in this Archipelago are mostly introduced species (Borges et al. 2022) and their presence might be very beneficial in these novel ecosystems, as these insects enhance the decomposition of dung, which can quickly accumulate in the pasturelands associated with cattle production.

Here, we present the diversity of dung beetles (Coleoptera, Scarabaeidae) associated with pasturelands in three Azorean islands (Faial, Pico and Terceira), collected within the project DUNGPOOL, which aims to evaluate the effects of the species pool, community assembly processes and increasing temperatures on the local diversity and ecosystem functions performed by dung beetles in island and mainland cattle-grazed pasturelands.

## General description

### Purpose

The main objective of this study is to provide a recent inventory of dung beetles species present in the pasturelands of Pico, Faial and Terceira Islands (Azores, Portugal).

### Additional information

The DUNGPOOL project was conceived to unravel how the size and composition of the regional species pool, the mechanisms of community assembly (including priority effects and biotic interactions) and near-term climate warming jointly shape dung beetle diversity and the key ecosystem functions they provide in pasturelands. By combining replicated field experiments on the Iberian mainland with parallel work on three mid-Atlantic islands (Pico, Faial and Terceira, Azores), the project exploits the natural contrast between a species-rich mainland pool and the species-poor, largely exotic island pools to test explicit biodiversity–ecosystem-function (BEF) hypotheses across spatial scales.

## Project description

### Title

Effects of species pool and community assembly processes on dung beetle diversity and ecosystem functions in a warming world (DUNGPOOL)

### Personnel

- Principal investigator: Ana M. C. Santos
- Fieldwork (site selection and experimental setting): Paulo A.V. Borges and Ana M. C. Santos.
- Fieldwork (authorisation): Azorean Regional Directorate for the Environment (Internationally Recognized Compliance Certificate CCIR-RAA/2024/6).
- Fieldwork team:
- Pico Island: Almudena Dueñas-Rojas, Eva Cuesta, Paulo A.V. Borges, Ana M. C. Santos
- Faial Island: Almudena Dueñas-Rojas, Eva Cuesta, Laurine Parmentier, Ana M. C. Santos
- Terceira Island: Almudena Dueñas-Rojas, Eva Cuesta, Abrão Leite, Ana M. C. Santos
- Taxonomist: Almudena Dueñas-Rojas and Eva Cuesta
- Darwin Core databases: Almudena Dueñas-Rojas

### Funding

Agencia Estatal de Investigación, Ministerio de Ciencia, Innovación y Universidades (Spain) (PID2021-122380NA-I00); MICIU/AEI/10.13039/501100011033; FEDER, UE.

## Sampling methods

### Study extent

This study was conducted from June to July 2024, in a total of 84 sites of three Azorean Islands: 29 in Pico Island, 29 in Faial Island and 26 in Terceira Island. Most of these sites correspond to cattle-grazed pasturelands.

### Sampling description

**Figure 1.**
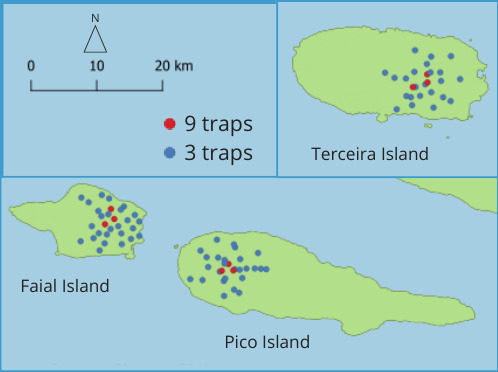
Sampling sites in three Azorean Islands (Portugal): Pico Island, Faial Island and Terceira Island. Blue dots correspond to sites sampled using three pitfall traps and red dots correspond to those sites in which nine pitfall traps were used.

In most sampling sites, we placed three pitfall traps filled with a soap-water solution and baited with fresh cow dung. However, in three of the sites in each island, we set nine baited pitfall traps (Fig. 1). Therefore, a total of 306 traps were placed, 105 in Pico, 105 in Faial and 96 in Terceira. The traps were collected after 72 hours of exposure.

The collected samples were stored in 70% alcohol. Then, in the laboratory they were cleaned and the dung beetles were separated. The specimens were then identified to the species level, labelled and preserved in 70% alcohol.

### Quality control

All specimens have been identified by taxonomists from Universidad Autónoma de Madrid (Eva Cuesta and Almudena Dueñas-Rojas).

## Geographic coverage

### Description

**Figure 2.**
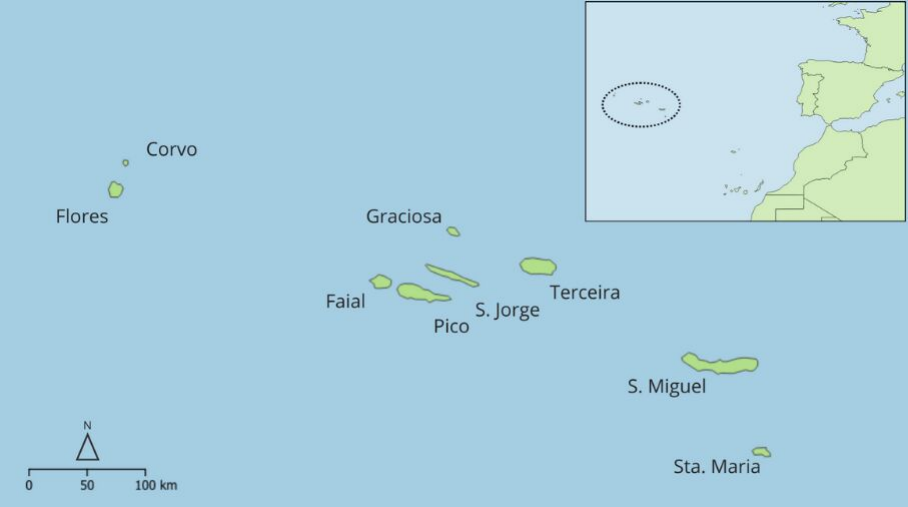
Location and map of the Azores Islands (Portugal): Pico (38.495833, -28.455833), Faial (38.588056, -28.665833) and Terceira (decimal coordinates 38.710556, -27.160000).

Three islands of the Archipelago of the Azores, Portugal: Pico, Faial and Terceira (Fig. 2). Most of the area of these islands is occupied by pastures used for cattle grazing (Borges et al. 2020).

### Coordinates

38.428000 and 38.793000 Latitude; -28.746000 and -27.054000 Longitude.

## Taxonomic coverage

### Taxa included

**Table:** 

Rank	Scientific Name	
phylum	Arthropoda	
class	Insecta	
order	Coleoptera	
family	Scarabaeidae	
subfamily	Aphodiinae	
subfamily	Scarabaeinae	

## Temporal coverage

**Data range:** 2024-6-27 – 2024-7-25.

## Usage licence

### Usage licence

Open Data Commons Attribution License

## Data resources

### Data package title

Dung beetles of Azorean cattle-grazed pasturelands – data of the DUNGPOOL project

### Resource link

https://doi.org/10.15470/ego4ut

### Alternative identifiers

https://www.gbif.org/dataset/cec13430-b81f-4d08-b066-5fd5e523aaa8

### Number of data sets

2

### Data set 1.

#### Data set name

Event Table

#### Data format

Darwin Core Archive format

#### Character set

UTF-8

#### Download URL

https://ipt.gbif.es/resource?r=dungpool_azores#anchor-downloads

#### Data format version

1.3

#### Description

The dataset was published in the Global Biodiversity Information Facility platform, GBIF (Dueñas-Rojas et al. 2025). The following data table includes all the records for which a taxonomic identification of the species was possible. The dataset submitted to GBIF is structured as a sample event dataset that has been published as a Darwin Core Archive (DwCA), which is a standardised format for sharing biodiversity data as a set of one or more data tables. The core data file contains 123 records (eventID).

**Data set 1.** 

Column label	Column description
EventID	Identifier of the events, unique for the dataset.
locationID	Identifier of the locations, unique for the dataset.
stateProvince	The name of the next smaller administrative region than country (state, province, canton, department, region etc.) in which the Location occurs.
islandGroup	The name of the island group in which the Location occurs (Azores Archipelago).
island	The name of the island on which the Location occurs (Pico, Faial or Terceira).
country	The full, unabbreviated name of the next smaller administrative region than stateProvince (county, shire, department etc.) in which the Location occurs (Portugal).
countryCode	The standard code for the country in which the Location occurs (PT).
municipality	The full, unabbreviated name of the next smaller administrative region than county (city, municipality etc.) in which the Location occurs.
locality	The specific description of the place.
habitat	The habitat for an Event.
minimunElevationinMetres	The lower limit of the range of elevation (altitude, usually above sea level), in metres.
decimalLatitude	Approximate centre point decimal latitude of the field site in GPS coordinates.
decimalLongitude	Approximate centre point decimal longitude of the field site in GPS coordinates.
geodeticDatum	Standard Global Positioning System coordinate reference for the location of the sample collection points.
coordinateUncertaintyinMetres	Uncertain value of coordinate metrics.
coordinatePrecision	Value in decimal degrees to a precision of six decimal places.
georeferenceSources	Navigation system used to record the location of sample collections.
samplingProtocol	The sampling protocol used to capture the species.
year	Year the sample was collected.
month	The integer month in which the Event occurred.
eventDate	The date-time or interval during which an Event occurred.
sampleSizeValue	A numeric value for a measurement of the size (time duration, length, area or volume) of a sample in a sampling Event.
sampleSizeUnits	The unit of measurement of the size (time duration, length, area or volume) of a sample in a sampling Event.
eventRemarks	Comments or notes about the Event.
type	The nature or genre of the resource.
modified	The most recent date-time on which the resource was changed.
language	A language of the resource.
licence	Reference to the licence under which the record is published.
datasetID	An identifier for the set of data.
datasetName	Name of the dataset.

### Data set 2.

#### Data set name

Occurrence Table

#### Data format

Darwin Core Archive format

#### Character set

UTF-8

#### Download URL

https://ipt.gbif.es/resource?r=dungpool_azores#anchor-downloads

#### Data format version

1.3

#### Description

The dataset was published in the Global Biodiversity Information Facility platform, GBIF (Dueñas-Rojas et al. 2025). The following data table includes all the records for which a taxonomic identification of the species was possible. The dataset submitted to GBIF is structured as an occurrence table that has been published as a Darwin Core Archive (DwCA), which is a standardised format for sharing biodiversity data as a set of one or more data tables. The core data file contains 130 records (occurrenceID).

**Data set 2.** 

Column label	Column description
eventID	Identifier of the events, unique for the dataset.
type	The nature or genre of the resource.
licence	Reference to the licence under which the record is published.
institutionCode	The code of the institution publishing the data.
institutionID	An identifier for the institution publishing the data.
datasetName	Name of the dataset.
basisOfRecord	The nature of the data record.
OcurrenceID	Identifier of the record, coded as a global unique identifier.
organismQuantity	A number or enumeration value for the quantity of Organisms.
organismQuantityType	The type of quantification system used for the quantity of organisms.
lifeStage	The age class or life stage of the Organism(s) at the time the Occurrence was recorded.
establishmentMeans	Statement about whether a Organism has been introduced to a given place and time through the direct or indirect activity of modern humans.
recordedBy	A list (concatenated and separated) of names of people, groups or organisations who performed the sampling in the field.
identifiedBy	A list of names of people, groups or organisations who assigned the Taxon to the subject.
dateIdentified	The date on which the subject was determined as representing the Taxon.
kingdom	Kingdom name.
phylum	Phylum name.
class	Class name.
order	Order name.
family	Family name.
subfamily	subfamily name.
scientificName	The full scientific name, with authorship and date information if known.
genus	Genus name.
specificEpithet	Specific epithet name.
scientificNameAuthorship	The authorship information for the scientificName formatted according to the conventions of the applicable nomenclaturalCode.
taxonRank	Lowest taxonomic rank of the record.
occurrenceStatus	A statement about the presence or absence of a taxon.

## Additional information

A total of 1141 dung beetle individuals were recorded across three Azorean Islands (Table 1): Pico (n = 18), Faial (n = 480) and Terceira (n = 643). These individuals were distributed amongst four species: *Calamosternus granarius* (Linnaeus, 1767) (Fig. 3a), *Labarrus lividus* (Olivier, 1789) (Fig. 3b), *Onthophagus taurus* (Schreber, 1759) (Fig. 3c) and *Onthophagus medius* (Kugelann, 1792) (Fig. 3d).

### Ecological and Biogeographical implications

The survey implemented in this study revealed a very impoverished dung beetle fauna, dominated by a single species, *Onthophagus taurus*. This dominance likely reflects both the species' high ecological plasticity and superior dispersal capabilities. In a recent study, Tonelli and Lobo (2025) concluded that species with enhanced dispersal abilities and greater ecological adaptability are more prone to succeed and establish stable populations in insular environments, where habitat diversity is often limited and ecological opportunities can be more restricted.

It is important to note, however, that the Azorean dung beetle assemblage is not limited to *O. taurus*. Other species have been recorded in the Archipelago, but were absent from our samples, such as *Onthophagus illyricus* (Scopoli, 1763) (Borges et al. 2022). The absence of *O. illyricus* in the present survey could be attributed to factors such as its lower abundance, more specific habitat requirements or possibly localised extinctions linked to habitat degradation or competition with more adaptable species like *O. taurus*. This observation aligns with broader patterns documented in island ecosystems, where a few highly adaptable species often dominate, leading to biotic homogenisation and a reduction in overall faunal diversity (Fernández-Palacios et al. 2021) and ecosystem functioning.

Moreover, after a recent revision of *O. vacca* complex, several authors concluded that a large number of past identifications of *Onthophagus vacca* (Linnaeus, 1767) belong to a different species, the very similar *Onthophagus medius* (Kugelann, 1792) (Rössner et al. 2010, Roy et al. 2016, Drăghici et al. 2019). Indeed, in Europe, two sibling species within the *Onthophagus vacca* complex can be reliably separated by a combination of information related with external morphology, seasonal timing and geographic range. Diagnostic characters are summarised in Table 1 of Rössner et al. (2010): *O. medius* typically bears larger, often confluent elytral spots and a complete basal pronotal margin, with a more truncate clypeal edge in males; in contrast, *O. vacca* has discrete, small black spots on the elytra, a pronotal basal line interrupted laterally and a sinuate male clypeal margin. Phenology further separates the two species, with adults of *O. vacca* emerging earliest in spring (activity peaking in April–May), whereas *O. medius* appears slightly later, with a narrower flight period peaking in May (Rössner et al. 2010). Biogeography also differs: *O. vacca* extends into northern Africa and is largely absent north of 50° N in central Europe, whereas *O. medius* occurs further north and at higher altitudes, but is absent from Mediterranean islands such as Corsica and Sardinia (Rössner et al. 2010). A careful re-examination of all Azorean material available in the University of Azores Insect Collection ("Dalberto Teixeira Pombo") with specimens collected by Borges (1989), Borges and Serrano (1989) and Borges et al. (2021) shows that all specimens, formerly cited as *O. vacca*, actually conform to *O. medius*. Moverover, we also examined more than 44 specimens recently collected in Terceira Island under the scope of an academic thesis (Mediavilla 2024) and the same applies. Therefore, possibly no genuine *O. vacca* have been detected in the Azores. Accordingly, we suggest that *Onthophagus medius* (Kugelann, 1792) is the sole valid member of the *O. vacca* complex in the Azorean fauna.

Notably, the general abundance of Azorean dung-beetle fauna is low and we may ask for the reasons for such pattern. In the Azores — based on 30 composite agricultural‐soil samples collected between 2018 and 2020 — organic‐contaminant loads are lower than in the other Macaronesian Archipelagoes, but still noteworthy in several categories (Acosta-Dacal et al. 2022). The one pharmaceutical‐active compound in Azorean soils that originates from veterinary treatment of cattle is oxfendazole, a broad‐spectrum anthelmintic commonly administered to cows. It was detected in 10% of the Azores samples, indicating its use in livestock and subsequent entry into soils via manure and pasture fertilisation (Acosta-Dacal et al. 2022). Modern livestock production almost universally relies on endectocides (e.g. ivermectin, moxidectin, doramectin) to control gastrointestinal nematodes, external parasites and flies. However, nothing is known in Azores about the consequences of modern livestock on coprophagous insects.

## Figures and Tables

**Figure 3a.**
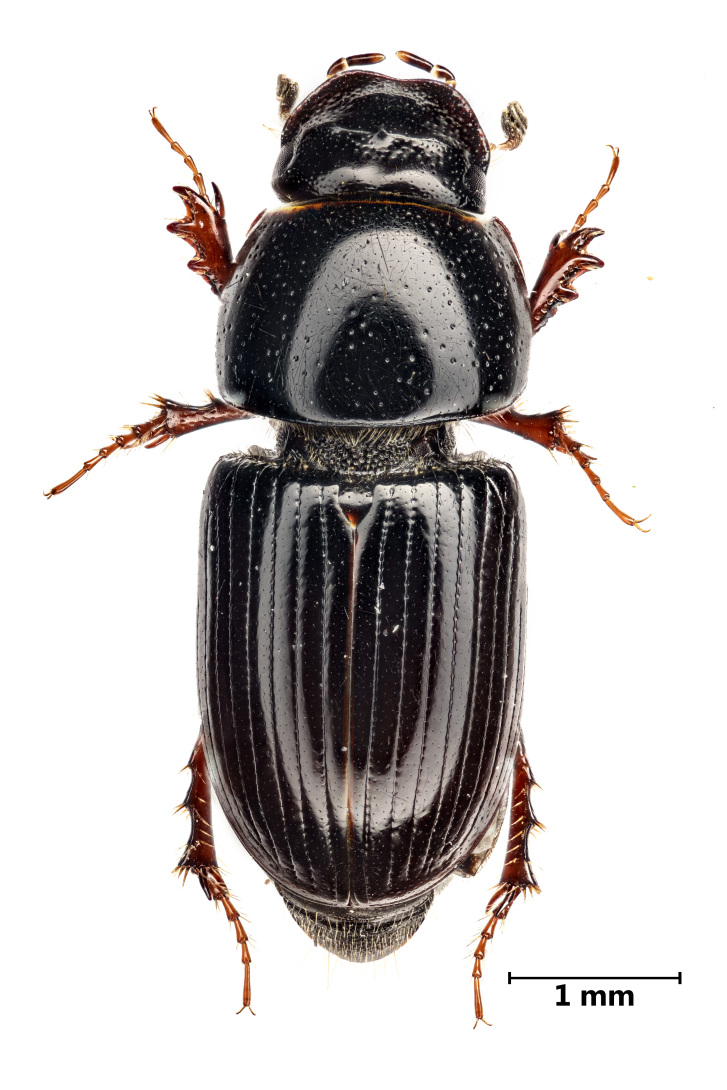
*Calamosternus granarius* (Linnaeus, 1767) (Credit: Javier Torrent, Azorean Biodiversity Group);

**Figure 3b.**
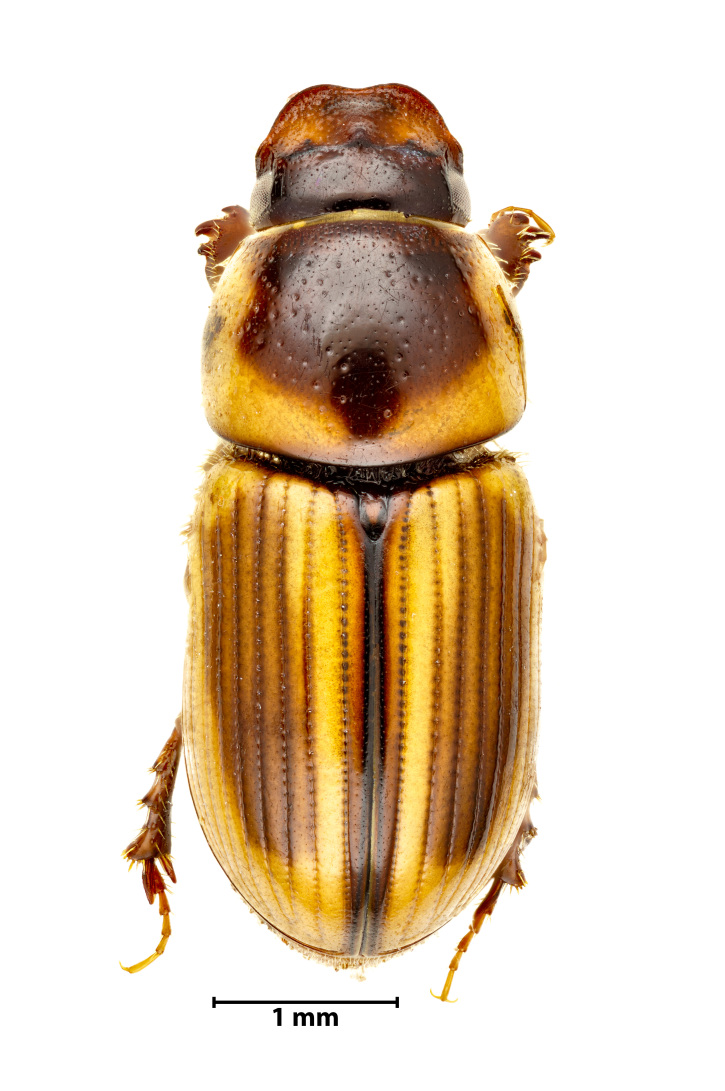
*Labarrus lividus* (Olivier, 1789) (Credit: Javier Torrent, Azorean Biodiversity Group);

**Figure 3c.**
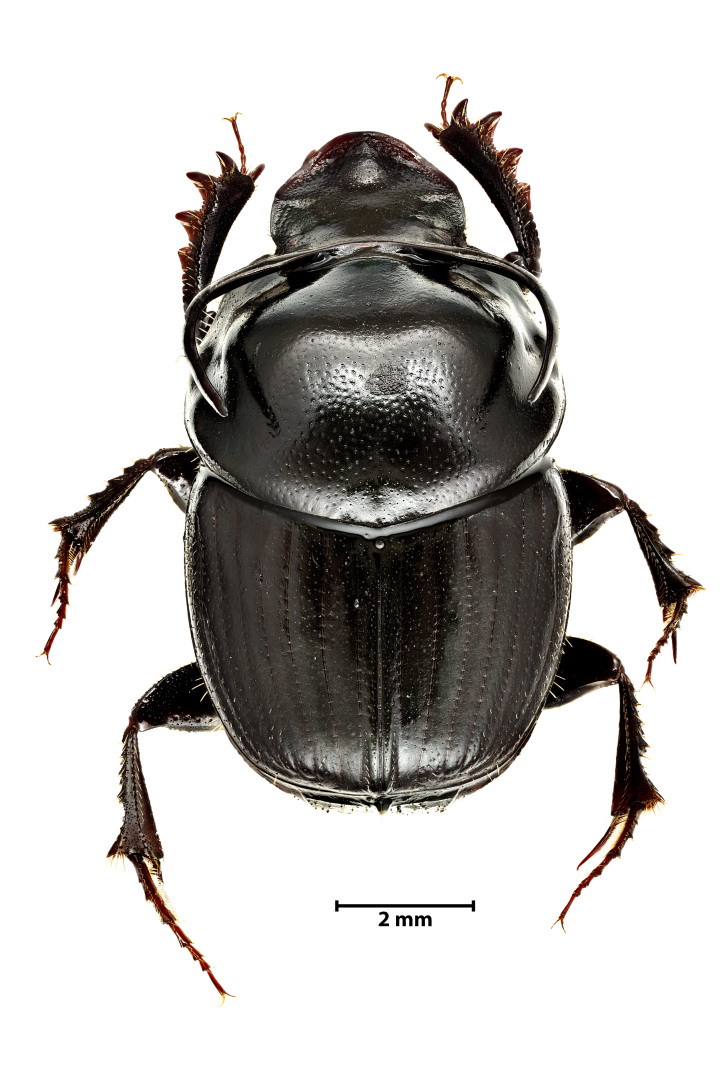
*Onthophagus taurus* (Schreber, 1759) (male) (Credit: Javier Torrent, Azorean Biodiversity Group);

**Figure 3d.**
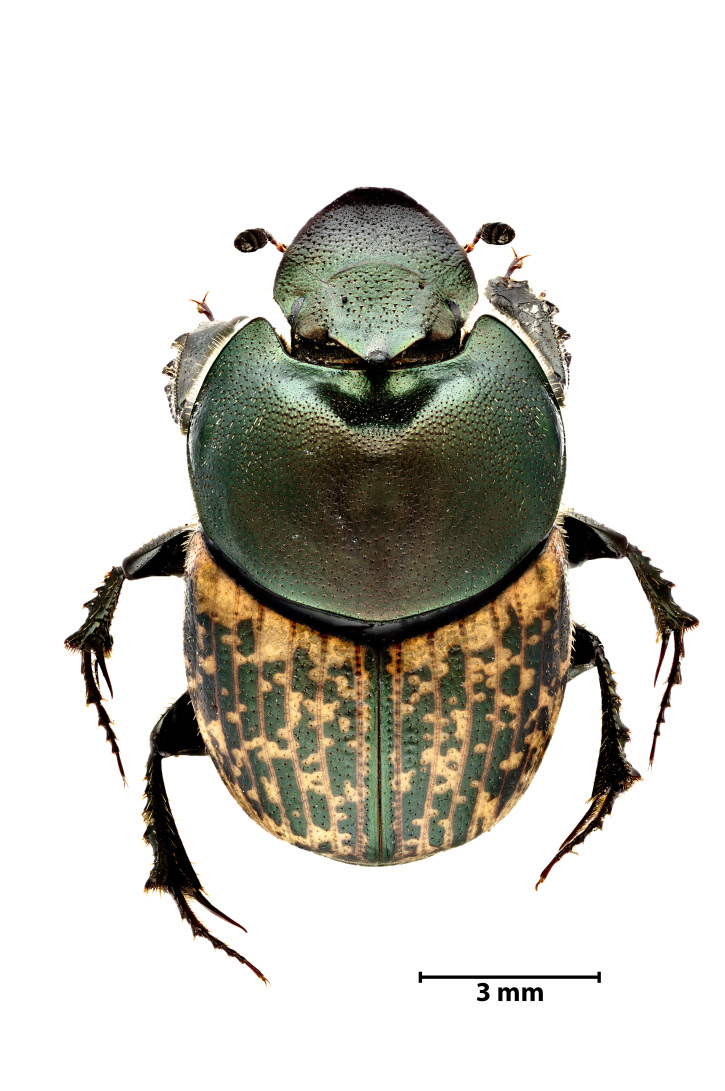
*Onthophagus medius* (Kugelann, 1792) (Credit: Javier Torrent, Azorean Biodiversity Group).

**Table 1.** List of species and number of individuals found on Pico Island (PIC), Faial Island (FAI) and Terceira Island (TER).

**Family**	**Subfamily**	**Scientific name**	**PIC**	**FAI**	**TER**
Scarabaeidae	Aphodiinae	*Calamosternus granarius* (Linnaeus, 1767)	1	0	1
Scarabaeidae	Aphodiinae	*Labarrus lividus* (Olivier, 1789)	0	2	33
Scarabaeidae	Scarabaeinae	*Onthophagus taurus* (Schreber, 1759)	17	478	606
Scarabaeidae	Scarabaeinae	*Onthophagus medius* (Kugelann, 1792)	0	0	3
